# Menopausal Symptoms among Breast Cancer Patients: A Potential Indicator of Favorable Prognosis

**DOI:** 10.1371/journal.pone.0075926

**Published:** 2013-09-30

**Authors:** Yong Chen, Tsogzolmaa Dorjgochoo, Ping-Ping Bao, Ying Zheng, Hui Cai, Wei Lu, Xiao-Ou Shu

**Affiliations:** 1 Department of Science & Education, Shanghai Municipal Center for Disease Control & Prevention, Shanghai, China; 2 Division of Epidemiology, Department of Medicine and Vanderbilt-Ingram Cancer Center, Vanderbilt University School of Medicine, Vanderbilt University, Nashville, Tennessee, United States of America; 3 Department of Cancer Prevention & Control, Shanghai Municipal Center for Disease Control & Prevention, Shanghai, China; 4 Department of Cancer Prevention & Control, Shanghai Institute of Preventive Medicine, Shanghai, China; Baylor College of Medicine, United States of America

## Abstract

Menopausal symptoms have been suggested to be an indicator of better prognosis among patients treated for breast cancer, because women who experience these symptoms usually have a lower level of estrogen. We tested this hypothesis in a population-based, prospective cohort study involving 4,842 women with stage 0 to III primary breast cancer who were enrolled in the Shanghai Breast Cancer Survival Study between March 2002 and April 2006, were aged 20 to 75 years, and were recruited 6 months post-diagnosis. They were followed-up by in-person surveys and record linkages with the vital statistics registry. Cox regression analysis was used to evaluate the association of menopausal symptoms at baseline with breast cancer recurrence. Approximately 56% of patients experienced at least one menopausal symptom, including hot flashes, night sweats, and/or vaginal dryness at baseline. During a median follow-up period of 5.3 years, 720 women had a recurrence. Experiencing hot flashes or having ≥2 menopausal symptoms was associated with lower risk of recurrence among premenopausal women (hazard ratio [HR]=0.77, 95% confidence interval [CI]: 0.62-0.96 for hot flashes; 0.73, 0.56-0.96 for ≥2 menopausal symptoms). Lower recurrence risk in relation to hot flashes was also observed among women who were not overweight/obese (HR=0.78, 95% CI: 0.64-0.99), those with relatively low waist-to-hip ratio (WHR) (HR=0.77, 95% CI: 0.61-0.97), and those who used tamoxifen (HR=0.75, 95% CI: 0.58-0.98). Consistently experiencing multiple menopausal symptoms was associated with lower recurrence risk among women with low WHR or who used tamoxifen. This large, population-based cohort study of women with breast cancer confirms that experiencing menopausal symptoms is an indicator of favorable breast cancer prognosis.

## Introduction

Breast cancer is the most common malignancy among women worldwide. Approximately 14% of all female cancer deaths are caused by breast cancer, and it is the second leading cause of cancer-related deaths among women [1]. The estimated age-adjusted incidence rate was 123.8 per 100,000 women per year, and the death rate was 22.6 per 100,000 women per year in 2006-2010 based on data from 18 SEER registries in the USA [2]. In China, the incidence of breast cancer was 21.21 per 100,000 and the mortality rate was 5.13 per 100,000 based on data from 104 cancer registries covering 85 million people in 2009 [3].

Women with breast cancer, particularly those who are younger, commonly experience menopausal symptoms after initial cancer treatment [4–6], including vasomotor (hot flashes and night sweats), atrophic (vaginal dryness), psychological (mood swings), cognitive (fatigue, sleep problems), and somatic (body pain) symptoms due to sudden estrogen withdrawal with the use of chemotherapy and anti-estrogenic treatments, such as tamoxifen [6,7]. In Western countries, most women experience hot flashes with natural menopause [8,9], and as many as 65%-78% of breast cancer patients experience menopausal symptoms after diagnosis and treatment [5,10,11]. Among women living in southeast Asia, it was reported that only about 10%-20% of women report experiencing hot flashes or having psychological complaints during natural transitions to menopause [12]. It has been suggested that this difference may be a result of higher soy food or soy isoflavone consumption among Asian women [13,14]. On the other hand, Asian-American women who experienced natural menopause have reported a high prevalence of vasomotor symptoms (up to 47% for hot flashes and 32% for sweating) [14]. In a recent study, we found a high prevalence of menopausal symptoms (56%-63%), primarily hot flashes and night sweats, among women in China treated for breast cancer, particularly among those who consumed high amounts of soy food [4,15].

Menopausal symptoms have serious effects on women’s quality of life, and medical intervention may be required to alleviate them [9]. This is particularly true for breast cancer patients because of the sudden and severe occurrence of such symptoms at the onset of cancer treatment, especially during chemotherapy and tamoxifen therapy [16–18]. It is generally accepted that estrogen plays a major role in breast cancer development and progression [19,20], suggesting that breast cancer patients with menopausal symptoms could have a better prognosis than those without menopausal symptoms [21] because of estrogen deprivation [22,23]. Mortimer et al. examined the association between the prevalence of menopausal symptoms with disease outcomes among women treated for breast cancer in the Women’s Healthy Eating and Living (WHEL) study in the US and found that women with hot flashes at baseline were less likely to develop disease progression, independent of age, tumor stage, and hormone receptor status, than those without symptoms [21]. To date, no study has investigated the potential role of menopausal symptoms as an indicator of breast cancer prognosis in Asian populations. In the present study, we examined the association of menopausal symptoms experienced by breast cancer patients with disease-specific outcomes such as recurrence in the Shanghai Breast Cancer Survival Study (SBCSS).

## Methods

### Ethics statement

The SBCSS was approved by the institutional review boards of Vanderbilt University and the Shanghai Municipal Center for Disease Control and Prevention. Before interviews were conducted, written informed consent was obtained from all patients.

### Study population

The SBCSS is a large, longitudinal, population-based study that recruited women aged 20-75 years who were diagnosed with primary breast cancer between March 2002 and April 2006. Breast cancer patients were identified from the population-based Shanghai Cancer Registry and recruited into the study approximately six months after cancer diagnosis. The inclusion criteria were: 1) diagnosis of primary breast cancer, 2) being a local resident and having a permanent household registration in Shanghai, China, and 3) alive at study recruitment. Initially, 6,299 patients were identified, of which 5,042 completed the baseline survey. Reasons for non-participation included: refusal (n=757), absence during study enrollment (n=258), unable to contact (n=83), and other miscellaneous reasons, such as health or communication problems (n=159). We excluded patients with distant metastases (TNM stage IV: n=28), because their treatment, clinical condition, and disease outcomes could affect their assessment of menopausal symptoms. Patients taking hormone replacement therapy (n=175) were excluded as well, since this therapy may affect the occurrence of menopausal symptoms. A total of 4,842 breast cancer patients remained for analysis.

### Data collection

In-person interviews were conducted by trained interviewers at 6 months after breast cancer diagnosis by using structured questionnaires to collect information on demographic characteristics, reproductive and medical history, lifestyle, diet, use of complementary and alternative medicine, and quality of life. Clinical information such as cancer stage at diagnosis, tumor estrogen receptor (ER) and progesterone receptor (PR) status, and primary treatments, including surgery, radiation therapy, chemotherapy, immune therapy, and tamoxifen use, were also collected. The medical record of each patient was reviewed to verify clinical and treatment information. Anthropometric measurements such as height, weight, and waist and hip circumferences were taken according to a standard protocol. Body-mass index (BMI) and waist-to-hip ratio (WHR) were calculated based on these measurements. History of chronic disease before breast cancer diagnosis was collected and coded according to the International Classification of Diseases Ninth Revision, Clinical Modification (ICD-9-CM) [24]; and a Charlson co-morbidity score was derived [25]. We used a validated food frequency questionnaire [26] to measure daily intakes of soy-containing foods. Estimates of soy isoflavone intake were calculated by summing the amount consumed of each soy-containing food and the isoflavone content of each food item according to values from the Chinese Food Composition Tables 2002 [27]. Menopausal status, defined as the cessation of menstruation for 12 months or longer, excluding lapses due to pregnancy or breastfeeding, was also determined at baseline. Menopausal symptoms were assessed at baseline, as reported previously [4]. Briefly, patients were queried about menopausal symptoms they had experienced according to standard criteria. If a patient had one or more of three typical symptoms, including hot flashes, night sweats, and vaginal dryness, since diagnosis and during treatment, she was then considered to be positive for menopausal symptom status. Participants were followed through in-person surveys conducted at 18, 36, and 60 months after cancer diagnosis (5.3 median years). Survival status was determined by periodic linkage with the Shanghai Vital Statistics Registry.

### Statistical Analysis

The demographic, clinical, and lifestyle characteristics of breast cancer patients with or without any of the three major menopausal symptoms were compared using the Cochran-Mantel-Haenszel test for categorical variables and analysis of variance for continuous variables. Breast cancer recurrence was the main outcome of this study. Recurrence included local/regional recurrence, distant recurrence/metastasis, or development of new primary breast cancer [28]. Disease-free survival time was defined as the time from breast cancer diagnosis to occurrence of a study event, censoring at the date of last in-person contact or August 4, 2011.

We used Cox proportional hazards models to evaluate associations of menopausal symptoms (with the presence or absence of any symptoms, each individual symptom, or the number of symptoms: 0, 1, or ≥2) with the risk of breast cancer recurrence by estimating hazard ratios (HRs) and 95% confidence intervals (CIs) using age as the time scale. Entry time was defined as age at diagnosis, and exit time was defined as age at recurrence or censoring at non-breast cancer death or last survey. Models were adjusted for demographic, lifestyle, and clinical prognostic factors, which included age at diagnosis (continuous); educational level (categories); number of pregnancies (0-1, 2 and ≥3); marital status (currently married/not married); history of chronic disease expressed as Charlson co-morbidity index (0/≥1); self-reported quality of life (poor, average, or good); received radiotherapy, chemotherapy, tamoxifen (where appropriate) or immunotherapy (yes/no); TNM stage (0-I, II, III, or missing); and tumor hormone receptor status (ER+/- and/or PR+/-). We examined associations of menopausal symptoms and risk of recurrence in relation to menopausal status (pre- vs. postmenopausal). Further, we examined joint associations for menopausal symptoms and tamoxifen use (yes *vs*. no), soy isoflavone intake (low: <36.8 vs. high: ≥36.8 mg/day, median intake), and overweight/obesity status (BMI <25.0 *vs*. ≥25.0 kg/m^2^ or WHR <0.832 *vs*. ≥0.832, median value) with breast cancer recurrence. Women who reported no menopausal symptoms or who had high BMI, high WHR, no use of tamoxifen, or low isoflavone intake were used as the reference group in these analyses. Multiplicative interactions between menopausal symptoms and these exposures were examined using the log likelihood ratio test, which compared the model including only the main effects with the model that included both the main effects and the interactive terms. The statistical significance level was set at *P* < 0.05 and all tests were two-sided. SAS software, version 9.2 (SAS Institute, Cary, NC) was used to perform the statistical tests.

## Results

During a median follow-up time of 5.3 years, 676 total deaths and 720 recurrences were reported. About 56% of all patients reported one or more menopausal symptom at baseline. Prevalence for hot flashes was 44.2%, for night sweats was 35.4%, and for vaginal dryness was 8.8%. Among patients with TNM 0-I breast cancer: 808 had hot flashes, 611 had night sweats, and 165 had vaginal dryness; among patients with TNM II breast cancer: 1058 had hot flashes, 870 had night sweats, and 205 had vaginal dryness; and among patients with TNM III breast cancer: 188 had hot flashes, 153 had night sweats, and 36 had vaginal dryness. Demographic, clinical, and lifestyle characteristics in relation to the presence or absence of any of the three major menopausal symptoms are presented in Table **1**. The mean (SD) age of women at breast cancer diagnosis was 53.3 (10.1) years; 241 (5.0%) women were less than 40 years old, 1980 (40.9%) aged 40-50 years, 1383 (28.6%) aged 50-60 years, and 1238 (25.5%) aged more than 60 years. Patients who were younger at diagnosis, had a middle and high school education, had fewer pregnancies, were married, were premenopausal, were lean according to BMI and WHR, had no history of chronic disease, had higher daily soy-isoflavone intake, had poor quality of life, received chemotherapy and/or immunotherapy, used tamoxifen, or had positive ER/PR status were more likely to experience menopausal symptoms (*P* <0.05 for all). Menopausal symptoms did not appear to vary by level of regular exercise, vitamin supplement use, drinking tea or alcohol, cigarette smoking, surgical treatment or radiotherapy, disease stage (TNM stage), or family history of breast cancer.

**Table 1:** Characteristics of breast cancer patients with and without menopausal symptoms at 6 months post-diagnosis, the Shanghai Breast Cancer Survival Study (SBCSS), n=4,842.

	**No Menopausal SymptomsN=2,133 (44.1%)**	**Any Menopausal Symptom^a^ N=2,709 (55.9%)**	***P^b^***
**Demographic characteristics**			
Age at diagnosis (y), mean ± SD	55.6 ± 11.4	51.5 ± 8.5	<0.01
Education,^c^%			
Primary school or no formal education	16.9	8.0	
Middle school	31.1	38.1	
High school	34.9	39.9	
College or above	17.1	14.0	<0.01
No. of Pregnancies, %			
0-1	22.4	25.4	
2	28.8	36.6	
≥3	48.8	38.0	<0.01
Marital status, %			
Married	83.8	90.6	
Other	16.2	9.4	<0.01
Menopausal status, %			
Premenopausal	38.9	60.0	
Postmenopausal	61.1	40.0	<0.01
**Lifestyle factors**			
Body-Mass Index (kg/m^2^), mean ± SD	24.3 ± 3.5	23.9 ± 3.4	<0.01
Waist-Hip Ratio, mean ± SD	0.836 ± 0.056	0.833 ± 0.053	0.02
Exercised regularly, %	64.5	64.8	0.82
Ever used vitamin supplements, %	27.7	29.8	0.11
Ever drank tea regularly, %	22.9	24.1	0.32
Ever smoked cigarettes regularly, %	2.7	2.7	0.90
Ever drank alcohol regularly, %	2.5	3.5	0.06
Isoflavone intake (mg/day), mean ± SD	44.7 ± 36.5	46.9 ± 38.9	0.04
Self-reported quality of life, %			
Poor	7.4	8.8	
Average	72.3	73.9	
Good	20.2	17.3	<0.01
**Clinical characteristics**			
Treatment, %			
Surgical treatment (mastectomy)	93.5	94.6	0.44
Radiotherapy	30.9	33.2	0.10
Chemotherapy	88.0	93.6	<0.01
Immunotherapy	12.4	16.4	<0.01
Tamoxifen use	45.8	57.2	<0.01
Disease stage (TNM), %			
0-I	35.9	36.9	
II	49.4	49.8	
III	10.2	8.7	
Unknown	4.5	4.6	0.73
Tumor hormone receptor status, %			
ER positive	60.8	66.0	<0.01
PR positive	54.6	60.8	<0.01
History of chronic disease, %^d^	22.5	17.9	<0.01
Family history of breast cancer, %	5.7	5.4	0.66

a Any menopausal symptom includes hot flashes, night sweats and/or vaginal dryness

b *P* values from ANOVA procedure for continuous variables and Cochran-Mantel-Haenszel statistics for categorical variables

c Typically, primary school: grade 1 to 5; middle school: grade 6 to 8; high school: grade 9-12. Before 1980, the middle school and high school could only take 2 years each.

d History of chronic disease: participants with a Charlson comorbidity index ≥1;

Abbreviations: ER, estrogen receptor; PR, progesterone receptor

**Figure 1:**
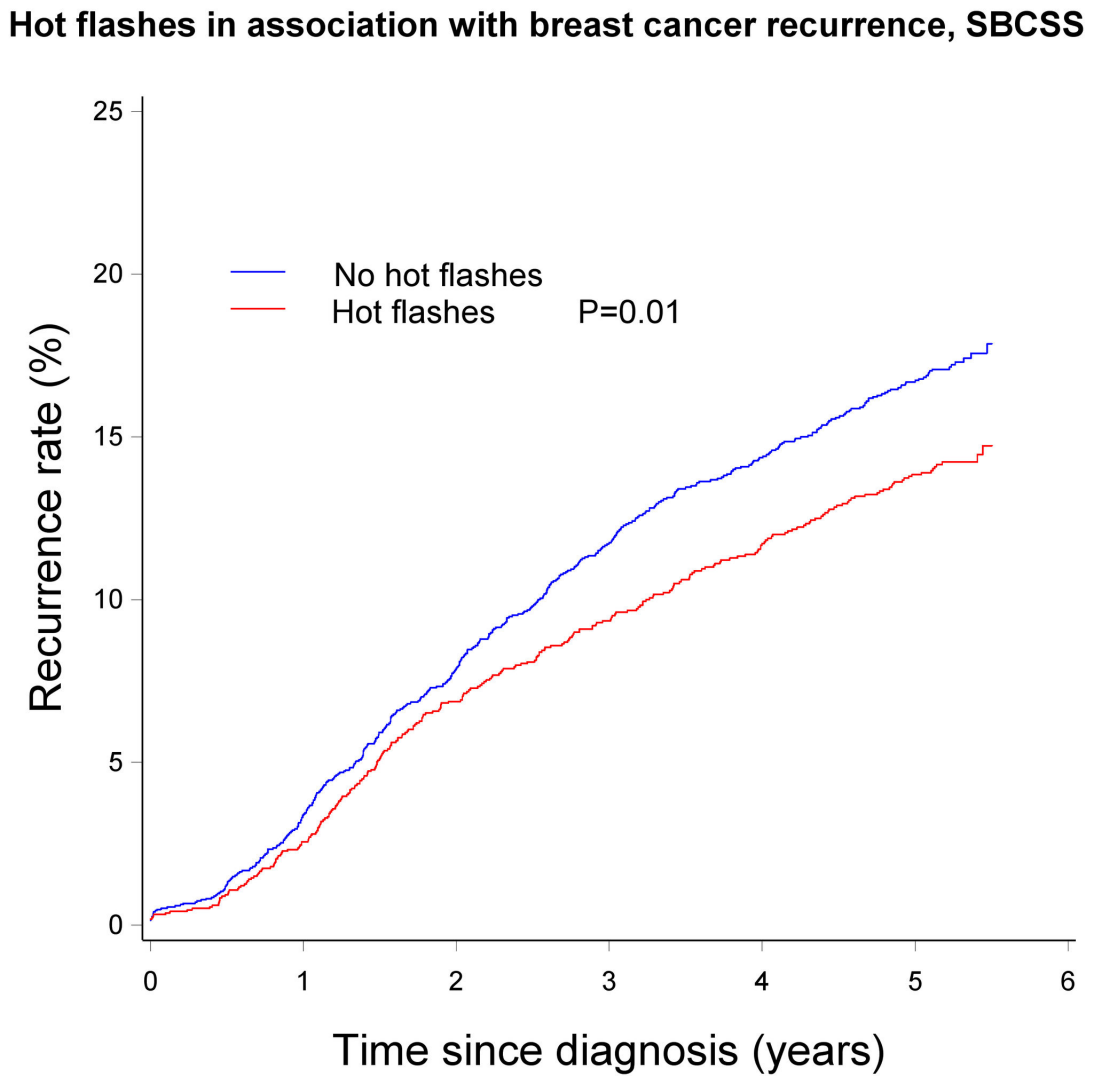
Hot flashes in association with breast cancer recurrence, SBCSS.

**Figure 2:**
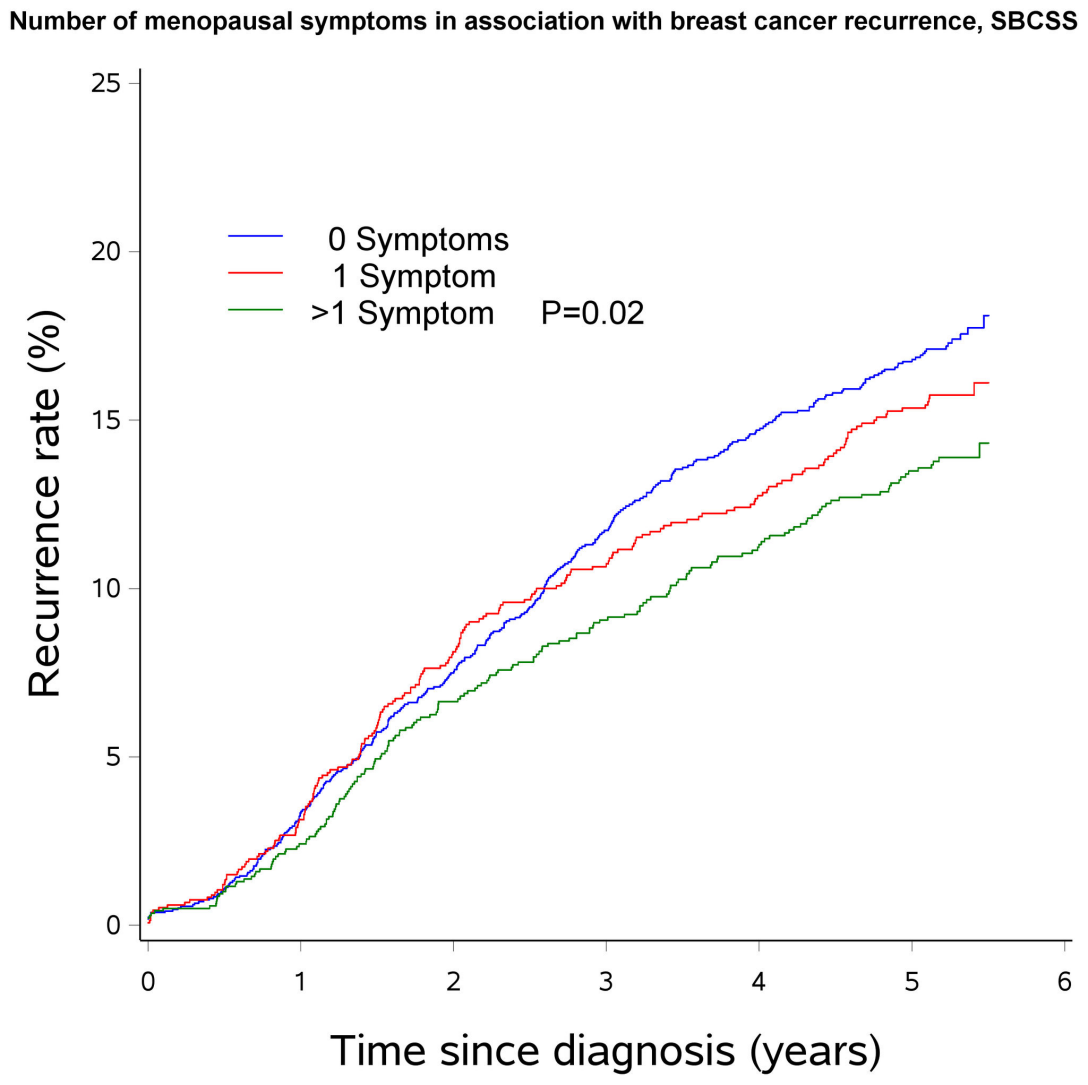
Number of menopausal symptoms in association with breast cancer recurrence, SBCSS.

Associations of breast cancer recurrence with menopausal symptoms according to menopausal status are presented in Table **2**. Premenopausal women who experienced hot flashes had a significantly lower risk of recurrence than those who did not have the symptom (HR=0.77, 95% CI: 0.62-0.96). Similarly, lower risk of recurrence was observed as the number of menopausal symptoms experienced increased among premenopausal women (HR= 0.73, 95% CI: 0.56-0.96 for experiencing ≥2 symptoms vs. for women without symptoms). No significant associations were found among postmenopausal women. Tests for multiplicative interaction between menopausal symptoms and menopausal status with recurrence risk, however, were not significant (*P* interactions >0.05 for all). Kaplan-Meier Curves describing the association of hot flashes and number of menopausal symptoms with recurrence is shown in Figures **1** and **2**.

**Table 2:** Postdiagnosis menopausal symptoms in association with breast cancer recurrence by menopausal status, SBCSS (n=4,842).

	**Menopausal status**							
Experience with menopausal symptoms	**Premenopausal, n=2,455**			**Postmenopausal, n=2,387**				
	Events	HR^a^ (95% CI)	HR^b^ (95% CI)	Events	HR^a^ (95% CI)	HR^b^ (95% CI)	*P* inter^a^	*P* inter^b^
**Any menopausal symptom^c^**								
No	126	1.00 (reference)	1.00 (reference)	218	1.00 (reference)	1.00 (reference)		
Yes	209	0.82 (0.65-1.03)	0.83 (0.66-1.04)	167	1.01 (0.82-1.25)	1.03 (0.83-1.27)	0.19	0.19
**Hot flashes**								
No	168	1.00 (reference)	1.00 (reference)	266	1.00 (reference)	1.00 (reference)		
Yes	167	0.77 (0.62-0.96)	0.78 (0.63-0.98)	119	1.01 (0.80-1.27)	1.03 (0.82-1.30)	0.11	0.10
**Night sweats**								
No	199	1.00 (reference)	1.00 (reference)	279	1.00 (reference)	1.00 (reference)		
Yes	136	0.88 (0.70-1.10)	0.88 (0.71-1.11)	106	1.00 (0.79-1.25)	1.00 (0.79-1.26)	0.48	0.51
**Vaginal dryness**								
No	306	1.00 (reference)	1.00 (reference)	357	1.00 (reference)	1.00 (reference)		
Yes	29	0.89 (0.61-1.32)	0.92 (0.62-1.35)	28	1.11 (0.75-1.64)	1.11 (0.75-1.65)	0.38	0.38
**No. of menopausal symptoms**								
0	126	1.00 (reference)	1.00 (reference)	218	1.00 (reference)	1.00 (reference)		
1	103	0.93 (0.71-1.21)	0.93 (0.71-1.22)	94	1.04 (0.81-1.34)	1.06 (0.82-1.36)		
≥2	106	0.73 (0.56-0.96)	0.74 (0.57-0.97)	73	0.97 (0.73-1.28)	0.98 (0.74-1.30)	0.27	0.28

a HR: Adjusted for age at diagnosis (continuous); education (categories); number of pregnancies (categories); marital status; history of chronic disease (Charlson comorbidity score 0/≥1); self-reported quality of life (categories); received radiotherapy, chemotherapy, or immunotherapy; TNM, ER and PR status and *P*-interaction^a^ across subgroups by menopausal status

b HR: Additionally adjusted for BMI (continuous), tamoxifen use, soy isoflavone intake (continuous) and *P*-interaction^b^ across subgroups by menopausal status

c Includes hot flashes, night sweats, and/or vaginal dryness

Note: Multivariate HRs and their corresponding 95% CIs were derived from Cox proportional hazards models, using age as the time scale in all analyses

The joint effects of menopausal symptoms, BMI, and WHR on breast cancer recurrence are presented in Tables **3** and **4**. We found that experiencing hot flashes was associated with a lower risk of recurrence among women with low BMI (HR= 0.78, 95% CI: 0.64-0.99) or low WHR (HR= 0.77, 95% CI: 0.61-0.97) compared with those without hot flashes and with high BMI (≥25.0) or with high WHR (≥0.832). However, test for multiplicative interaction was only significant for WHR (*P*-interaction=0.05), but not for BMI (*P-*interaction=0.24). Similarly, experiencing ≥2 menopausal symptoms was associated with a lower risk of recurrence among women with low BMI (HR= 0.81, 95% CI: 0.62-1.05) or low WHR (HR= 0.71, 95% CI: 0.53-0.95), but not among women with high body or abdominal fatness and without menopausal symptoms. Interaction tests between the number of menopausal symptoms and BMI or WHR were not statistically significant (*P-*interaction=0.51 and 0.13, respectively).

**Table 3:** Postdiagnosis menopausal symptoms in association with breast cancer recurrence by body-mass-index (BMI), SBCSS (n=4,842).

	**BMI** (universal cutoff point)				
Experience with menopausal symptoms	**Overweight/obese (≥25 kg/m^2^) n=1,705**		**Not overweight/obese (<25 kg/m^2^) n=3,137**		
	cases	HR^a^ (95% CI)	cases	HR^a^ (95% CI)	*P* interaction
**Any menopausal symptom^b^**					
No	815	1.00 (reference)	1318	0.98 (0.78-1.22)	
Yes	890	1.05 (0.83-1.33)	1819	0.84 (0.67-1.04)	0.18
**Hot flashes**					
No	1035	1.00 (reference)	1669	0.95 (0.78-1.15)	
Yes	670	1.01 (0.79-1.29)	1468	0.78 (0.64-0.99)	0.24
**Night sweats**					
No	1127	1.00 (reference)	1999	0.88 (0.73-1.07)	
Yes	578	0.95 (0.74-1.22)	1138	0.82 (0.66-1.03)	0.90
**Vaginal dryness**					
No	1574	1.00 (reference)	2841	0.88 (0.75-1.03)	
Yes	131	0.97 (0.61-1.54)	296	0.86 (0.61-1.22)	0.97
**No. of menopausal symptoms**					
0	815	1.00 (reference)	1318	0.98 (0.78-1.22)	
1	455	1.20 (0.92-1.58)	870	0.85 (0.66-1.11)	
≥2	435	0.88 (0.65-1.20)	949	0.81 (0.62-1.05)	0.51

a HRs are adjusted for age at diagnosis (continuous), education (categories), number of pregnancies (categories), marital status, history of chronic disease (Charlson comorbidity score 0/≥1), perceived quality of life (categories), treatment received (radiotherapy, chemotherapy, immunotherapy, and/or tamoxifen; yes/no), TNM, and ER and PR status

b Included hot flashes, night sweats, and/or vaginal dryness

**Table 4:** Postdiagnosis menopausal symptoms in association with breast cancer recurrence by waist-to-hip ratio (WHR), SBCSS (n=4,842).

	**WHR** (median)				
Experience with menopausal symptoms	**High (≥0.832) n=2,428**		**Low (<0.832) n=2,414**		
	cases	HR^a^ (95% CI)	cases	HR^a^ (95% CI)	*P* interaction
**Any menopausal symptom^b^**					
No	1102	1.00 (reference)	1031	0.99 (0.79-1.24)	
Yes	1326	1.00 (0.82-1.24)	1383	0.83 (0.66-1.03)	0.22
**Hot flashes**					
No	1387	1.00 (reference)	1317	1.02 (0.83-1.24)	
Yes	1041	1.03 (0.84-1.27)	1097	0.77 (0.61-0.97)	0.05
**Night sweats**					
No	1572	1.00 (reference)	1554	0.92 (0.76-1.11)	
Yes	856	0.96 (0.78-1.09)	860	0.82 (0.65-1.04)	0.65
**Vaginal dryness**					
No	2246	1.00 (reference)	2169	0.91 (0.77-1.07)	
Yes	182	1.08 (0.74-1.57)	245	0.8 1 (0.54-1.20)	0.49
**No. of menopausal symptoms**					
0	1102	1.00 (reference)	1031	0.99 (0.79-1.24)	
1	651	1.05 (0.82-1.33)	674	0.93 (0.72-1.21)	
≥2	675	0.96 (0.75-1.23)	709	0.71 (0.53-0.95)	0.13

a HRs are adjusted for age at diagnosis (continuous), education (categories), number of pregnancies (categories), marital status, history of chronic disease (Charlson comorbidity score 0/≥1), perceived quality of life (categories), treatment received (radiotherapy, chemotherapy, immunotherapy, and/or tamoxifen; yes/no), TNM, and ER and PR status

b Included hot flashes, night sweats, and/or vaginal dryness

We also examined joint associations between menopausal symptoms and tamoxifen use or soy-isoflavone intake and breast cancer recurrence (Tables **5** and **6**). Tamoxifen users with hot flashes had a significantly lower risk of recurrence (HR= 0.75, 95% CI: 0.58-0.98) than those who did not use tamoxifen and reported no hot flashes. However, the interaction test across these subgroups was not significant (*P*-interaction=0.30). Similarly, patients who experienced ≥2 menopausal symptoms and used tamoxifen had a lower risk of recurrence (HR=0.71, 95% CI: 0.52-0.96) than those with no tamoxifen use and no menopausal symptoms. Associations between menopausal symptoms and breast cancer recurrence did not differ by soy-isoflavone intake.

**Table 5:** Menopausal symptoms in association with breast cancer recurrence by tamoxifen use, SBCSS (n=4,842).

	**Tamoxifen**				
Experience with menopausal symptoms	**Did not use n=2,314**		**Used^a^ n=2,093**		
	cases	HR^b^ (95% CI)	cases	HR^b^ (95% CI)	*P* interaction
**Any menopausal symptom^c^**					
No	1154	1.00 (reference)	791	0.79 (0.60-1.04)	
Yes	1160	0.92 (0.75-1.13)	1302	0.78 (0.60-1.01)	0.82
**Hot flashes**					
No	1439	1.00 (reference)	1029	0.86 (0.67-1.10)	
Yes	875	0.96 (0.78-1.19)	1064	0.75 (0.58-0.98)	0.30
**Night sweats**					
No	1571	1.00 (reference)	1279	0.77 (0.61-0.98)	
Yes	732	0.88 (0.71-1.10)	814	0.81 (0.61-1.06)	0.59
**Vaginal dryness**					
No	2108	1.00 (reference)	1908	0.83 (0.67-1.02)	
Yes	206	0.96 (0.67-1.37)	185	0.70 (0.42-1.17)	0.58
**No. of menopausal symptoms**					
0	1154	1.00 (reference)	791	0.79 (0.60-1.14)	
1	585	0.98 (0.77-1.24)	621	0.86 (0.63-1.17)	
≥2	575	0.85 (0.66-1.10)	681	0.71 (0.52-0.96)	0.75

a Tamoxifen use among ER+ patients only

b HRs are adjusted for age at diagnosis (continuous), education (categories), number of pregnancies (categories), marital status, history of chronic disease (Charlson comorbidity score 0/≥1), perceived quality of life (categories), treatment received (radiotherapy, chemotherapy, and/or immunotherapy; yes/no), TNM, and ER (where appropriate) and PR status

c Included hot flashes, night sweats, and/or vaginal dryness

**Table 6:** Menopausal symptoms in association with breast cancer recurrence by soy isoflavone intake, SBCSS (n=4,842).

	**Soy isoflavone intake** (median)				
Experience with menopausal symptoms	**Low (<36.8 mg/d) n=2,409**		**High (≥36.8 mg/d) n=2,433**		
	cases	HR^a^ (95% CI)	cases	HR^a^ (95% CI)	*P* interaction
**Any menopausal symptom^b^**					
No	1094	1.00 (reference)	1039	1.08 (0.87-1.34)	
Yes	1315	0.96 (0.77-1.20)	1394	0.96 (0.77-1.18)	0.59
**Hot flashes**					
No	1364	1.00 (reference)	1340	1.11 (0.91-1.34)	
Yes	1045	0.98 (0.78-1.22)	1093	0.91 (0.73-1.13)	0.26
**Night sweats**					
No	1589	1.00 (reference)	1537	1.04 (0.87-1.24)	
Yes	820	0.94 (0.75-1.18)	896	0.96 (0.78-1.20)	0.95
**Vaginal dryness**					
No	2205	1.00 (reference)	2210	1.00 (0.86-1.17)	
Yes	204	0.77 (0.49-1.20)	223	1.16 (0.82-1.64)	0.15
**No. of menopausal symptoms**					
0	1094	1.00 (reference)	1039	1.08 (0.87-1.34)	
1	650	1.04 (0.80-1.35)	675	1.03 (0.80-1.32)	
≥2	665	0.88 (0.68-1.15)	719	0.88 (0.68-1.14)	0.62

a HRs are adjusted for age at diagnosis (continuous), education (categories), number of pregnancies (categories), marital status, history of chronic disease (Charlson comorbidity score 0/≥1), perceived quality of life (categories), treatment received (radiotherapy, chemotherapy, and/or immunotherapy; yes/no), TNM, and ER (where appropriate) and PR status

b Included hot flashes, night sweats, and/or vaginal dryness

## Discussion

In this large population-based cohort study of 4,842 breast cancer patients aged 20-75 years, experiencing menopausal symptoms, especially hot flashes, as well as experiencing multiple symptoms during the first 6 months post-diagnosis, were associated with a lower risk of recurrence. These associations were predominantly seen among premenopausal women, women with low BMI or WHR, and those who used tamoxifen. To our knowledge, this study is the first large-scale, prospective investigation to provide evidence for an inverse association between menopausal symptoms, particularly hot flashes, shortly after breast cancer diagnosis and during the initial cancer treatment period, and breast cancer recurrence in Chinese women. Vaginal dryness and night sweats were less common among our study participants and were not associated with recurrence risk in our population, as has been previously reported for women in North America [23].

Although the biological mechanisms underlying vasomotor symptoms such as hot flashes and night sweats are not very clear, it has been reported that these symptoms may be related to the acute drop in estrogen levels during natural and medically induced menopause [22,29]. Women of reproductive age or premenopausal women usually do not experience these symptoms [9]. However, premenopausal women with breast cancer experience frequent and severe vasomotor symptoms as a result of chemotherapy and tamoxifen therapy [5,16,23]. Thus, having vasomotor symptoms following treatment for breast cancer may indicate estrogen deprivation or decreased levels of circulating estrogen, suggesting treatment efficacy [21,30]. This hypothesis is supported by our current findings that premenopausal women who experienced hot flashes following cancer treatment had a lower risk of breast cancer recurrence.

Tamoxifen is one of the most widely used drugs for breast cancer patients [31,32] and effectively reduces death and recurrence from breast cancer. Tamoxifen and its metabolites, particularly 4-hydroxy-tamoxifen (4-HO tamoxifen) and 4-hydroxy-*N*-desmethyl tamoxifen (endoxifen), have potent anticancer effects, because of their anti-estrogenic properties [31]. In the WHEL study, among 864 tamoxifen-using patients (aged 18-70 years) with Stage I-IIIa breast cancer, those who experienced hot flashes during the first 2-48 months after cancer treatment were less likely to develop recurrent breast cancer after 7.3 years than those who did not experience this symptom (12.9% vs. 21%, *P*=0.01) [21]. The authors conclude that hot flashes are a strong predictor of breast cancer-specific outcomes [21]. Consistent with the WHEL finding, we found that women treated with tamoxifen had a lower risk of recurrence only if they experienced hot flashes. It is likely that this group of women may have experienced very low estrogen levels and, thus, had more serious symptoms. Unfortunately, we did not collect information on the severity of symptoms, nor did we measure estrogen levels.

We also observed that patients with lower BMI or WHR had a lower risk of recurrence, if they had hot flashes or reported multiple symptoms. Higher BMI and WHR, indicators for overall and central obesity, have been associated with high circulating estrogen levels [33,34], which, in turn, have been implicated in breast cancer development and progression [35,36]. Women with low BMI or WHR, particularly those with menopausal symptoms, are likely to have lower estrogen or insulin levels, which could translate into lower risk for breast cancer recurrence and better prognosis.

Soy-isoflavone is known to have anti-estrogenic properties [37]. The association between soy food intake and menopausal symptoms among breast cancer patients has been described in detail in another publication, in which we found that women in the highest quartile of soy food intake were 159% more likely to experience hot flash than those in the lowest quartile of soy food intake at 36 months postdiagnosis [15]. Thus, breast cancer patients with high soy food consumption may have a lower level of estrogen exposure. We evaluated the interaction between soy food intake and menopausal symptoms in relation to breast cancer recurrence and found no evidence of interaction.

Menopausal symptoms are known to negatively affect women’s quality of life (QoL), including breast cancer survivors [38–40]. Our finding that experiencing menopausal symptoms was associated with a favorable outcome highlights the potential risk of alleviating menopausal symptoms by increasing estrogen level among breast cancer patients. More research on alternative, non-estrogen related approaches for reducing menopausal symptoms among breast cancer survivors are warranted.

Several potential limitations should be considered in this study. First, data on menopausal symptoms were collected through in-person interviews by self-reports. Some Chinese women may not report certain symptoms, such as vaginal dryness, considering it too private. Thus, misreporting and under-reporting are likely, which would have biased our results towards null. Second, we did not collect information on the severity, frequency, or duration of symptoms. Third, although the results were adjusted for a wide range of covariates, the possibility of residual confounding still exists. Fourth, the lack of direct measurement of estrogen hormone levels prevented us from investigating the underlying mechanisms for the associations between menopausal symptoms and breast cancer recurrence. Our study also has several strengths. This is a large, population-based, prospective study specifically designed to investigate the association of menopausal symptoms with breast cancer recurrence among Chinese women. The high response rates, as well as the detailed information on socio-demographic, anthropometric, clinical, and lifestyle characteristics, collected using a well-validated questionnaire, minimized selection bias and allowed us to account for confounding from multiple factors and to evaluate potential interactions.

In summary, we found that the occurrence of menopausal symptoms, including hot flashes, night sweats, and/or vaginal dryness during the first 6 months post-diagnosis and post- treatment, were associated with a lower risk of breast cancer recurrence among Chinese women. Although experience of menopausal symptoms is a subjective feeling from patients, the link between low estrogen exposure and menopausal symptoms has been well established [15]. Thus, experiencing hot flashes or multiple menopausal symptoms may be a clinically relevant predictor of better breast cancer outcomes, particularly for premenopausal women, tamoxifen users, and those with low body fatness when direct measurement of estrogen level is not available. Further research is needed to examine the relationship between the severity, frequency, and duration of menopausal symptoms and breast cancer outcomes, while accounting for other estrogen-related factors.
